# Impact of Minimal Residual Disease, Detected by Flow Cytometry, on Outcome of Myeloablative Hematopoietic Cell Transplantation for Acute Lymphoblastic Leukemia

**DOI:** 10.1155/2014/421723

**Published:** 2014-03-23

**Authors:** Merav Bar, Brent L. Wood, Jerald P. Radich, Kristine C. Doney, Ann E. Woolfrey, Colleen Delaney, Frederick R. Appelbaum, Ted A. Gooley

**Affiliations:** ^1^Division of Clinical Research, Fred Hutchinson Cancer Research Center, P.O. Box 19024, Seattle, WA 98109, USA; ^2^Department of Medicine, University of Washington, Seattle, WA 98195, USA; ^3^Department of Pathology, University of Washington, Seattle, WA 98195, USA; ^4^Department of Pediatrics, University of Washington, Seattle, WA 98195, USA; ^5^Department of Biostatistics, University of Washington, Seattle, WA 98195, USA

## Abstract

In this retrospective study, we evaluated the impact of pre- and posttransplant minimal residual disease (MRD) detected by multiparametric flow cytometry (MFC) on outcome in 160 patients with ALL who underwent myeloablative allogeneic hematopoietic cell transplantation (HCT). MRD was defined as detection of abnormal B or T cells by MFC with no evidence of leukemia by morphology (<5% blasts in marrow) and no evidence of extramedullary disease. Among 153 patients who had pre-HCT flow data within 50 days before transplant, MRD pre-HCT increased the risk of relapse (hazard ratio (HR) = 3.64; 95% confidence interval (CI), 1.87–7.09; *P* = .0001) and mortality (HR = 2.39; 95% CI, 1.46–3.90, *P* = .0005). Three-year estimates of relapse were 17% and 38% and estimated 3-year OS was 68% and 40% for patients without and with MRD pre-HCT, respectively. 144 patients had at least one flow value post-HCT, and the risk of relapse among those with MRD was higher than that among those without MRD (HR = 7.47; 95% CI, 3.30–16.92, *P* < .0001). The risk of mortality was also increased (HR = 3.00; 95% CI, 1.44–6.28, *P* = .004). These data suggest that pre- or post-HCT MRD, as detected by MFC, is associated with an increased risk of relapse and death after myeloablative HCT for ALL.

## 1. Introduction

Allogeneic hematopoietic cell transplantation (allo-HCT) is a potential curative treatment for children and adults with recurrent or high-risk acute lymphoblastic leukemia (ALL). However, relapse occurs in approximately 30% of patients with ALL after HCT [1, 2], with a relapse rate of more than 60% in high-risk patients [3]. The outcome of patients who relapse after HCT is usually poor despite salvage therapies. Outcome of ALL patients after HCT might be improved by identification of markers to predict impending relapse. Minimal residual disease (MRD) assessment relies on the identification of specific molecular or immunophenotypic markers on the leukemia cells. PCR is used to detect leukemia-specific fusion transcripts (e.g., BCR-ABL) or clone specific immunoglobulin (Ig) or T-cell receptor (TCR) genes. MRD detected by PCR has been demonstrated as an independent risk factor for ALL relapse after induction or consolidation therapy [4–7]. A number of studies have also shown clinical significance of MRD, as detected by PCR, in the transplant setting [8–16].

PCR methods for detection of MRD have high sensitivity, but are relatively labor intensive and not widely available. An alternative method for MRD measurement is by multiparameter flow cytometry (MFC), based on the detection of leukemia associated immunophenotypes that can be used to distinguish them from normal hematopoietic cells [17]. Using ≥4-color flow cytometry, leukemia-associated immunophenotypes can be identified in more than 90% of ALL patients, with detection limits of 10^−3^-10^−4^ [18–25]. Increasing evidence has demonstrated the prognostic importance of MRD detected by MFC in pediatric and adult patients with ALL in the nontransplant setting [18, 21, 24, 26–28]. Results have indicated that patients with detectable MRD by MFC at the end of induction and during maintenance therapy have a high rate of relapse. However, only a few studies have evaluated the clinical impact of MRD monitoring by MFC in ALL patients who undergo HCT [29, 30].

In the present study, we evaluated the value of MRD, detected by seven-color MFC before and after allo-HCT, in 160 pediatric and adult patients with ALL, to identify the impact of pre- and post-HCT MRD on relapse and survival posttransplant.

## 2. Patients and Methods

### 2.1. Study Cohort

Patients of all ages, identified from the Fred Hutchinson Cancer Research Center computerized database, were included in this retrospective study. Data were extracted from the transplantation database and from individual chart review. The study cohort included 160 patients, who underwent allo-HCT for treatment of ALL in remission (<5% blasts in marrow by morphology and no evidence of extramedullary disease) from April 2006 through March 2011. Patients received high-intensity conditioning regimens before HCT according to a standard treatment plan or prospective clinical trials at the Fred Hutchinson Cancer Research Center. 142 patients (89%) received TBI based conditioning, and the rest received regimens consisted of Treosulfan and Fludarabine, Busulfan and Cyclophosphamide, or Busulfan and Fludarabine. All patients provided informed consent for treatment according to transplantation protocols approved by the institutional review board. In addition, separate institutional approval was obtained to gather data from patient records and databases. The database was locked as of March 2013.

### 2.2. MFC Detection of MRD

MFC was performed on bone marrow aspirates as previously described [17, 31]. For B-lineage ALL, the panel consisted of one tube as follows: CD20-fluorescein isothiocyanate (FITC), CD10-Phycoerythrin (PE), CD34-PerCP-Cy5.5, CD19-PE-Cy7, CD38-Alexa 594 (A594), CD58-allophycocyanin (APC), and CD45-APC-H7. For T-lineage ALL, the panel consisted of one tube as follows: CD8 v450, CD2 FITC, CD5 PE, CD34 PE-TR, CD56 PE-Cy5, CD3 PE-Cy7, CD4 A594, CD7 APC, CD30 APC-A700, and CD45 APC-H7. For biphenotypic leukemia (two patients), the same combination of reagents used for B or T cell ALL was used and supplemented by a combination directed at abnormal myeloid progenitors ((1) HLA-DR PB, CD15 FITC, CD33 PE, CD19 PE-TR, CD117 PE-Cy5, CD13 PE-Cy7, CD38 A594, CD34 APC, CD71 APC-A700, CD45 APC-Cy7, (2) HLA-DR PB, CD64 FITC, CD123 PE, CD4 PE-TR, CD14 PE-Cy5.5, CD13 PE-Cy7, CD38 A594, CD34 APC, CD16 APC-A700, CD45 APC-Cy7, and (3) CD56 A488, CD7 PE, CD5 PE-Cy5, CD33 PE-Cy7, CD38 A594, CD34 APC, CD45 APC-Cy7). All antibodies were obtained from Beckman-Coulter (Fullerton, CA) or Becton Dickinson (BD Biosciences, San Jose, CA). Up to 1 million events per tube were acquired on a custom-built LSR II flow cytometer (BD Biosciences), and data compensation and analysis were performed by using noncommercial software developed in our laboratory. MRD was identified as a cell population showing deviation from the normal patterns of antigen expression seen on specific cell lineages at specific stages of maturation compared with either normal or regenerating marrow. When identified, the abnormal population was quantified as a percentage of the total white blood cell events. The software used to help analyzing MFC MRD in this study is WoodList version 2.7.7, the software used in the clinical hematopathology laboratory in our institution. Any level of residual disease was considered MRD-positive. Overt relapse was defined as >5% blasts by morphology or evidence of extramedullary disease.

### 2.3. Statistical Analyses

The hazards of relapse and overall mortality were compared between those with MRD and those without MRD using Cox regression. Patients were categorized as MRD-positive or MRD-negative pre-HCT based on the flow value closest to but before date of transplant, while post-HCT flow values were modeled as a time-dependent covariate. The association of pre-HCT MRD with outcome was assessed separately from the association of post-HCT MRD with outcome. Patients were also categorized based on pre- and post-HCT MRD as positive/positive, positive/negative, negative/negative, or negative/positive. This variable was modeled as a time-dependent covariate based on the status of the post-HCT MRD. Estimates of the probability of relapse were summarized by cumulative incidence estimates with death without relapse treated as a competing risk. Estimates of the probability of survival and relapse-free survival were obtained using the method of Kaplan and Meier.

## 3. Results

### 3.1. Patient Characteristics

The study cohort included 160 patients (62 pediatric and 98 adult, 99 males and 61 females), who underwent myeloablative allogeneic-HCT at the Fred Hutchinson Cancer Research Center from April 2006 to March 2011 for the treatment of ALL in remission (<5% blasts in marrow by morphology and no evidence of extramedullary disease). All patients had less than 5% bone marrow blasts and had no evidence of extramedullary disease and thus met the criterion for CR. Median age was 24.6 years (range, 0.6–61.8 years). There were 134 patients with B-ALL, 24 patients with T-ALL, and two patients with Biphenotypic Leukemia (one patient had markers of B cells and myeloid cells, and one patient had markers of T cells and myeloid cells). Ninety-nine patients had normal karyotype; 31 patients had Ph+ ALL as defined by conventional cytogenetics, FISH, or PCR; 15 patients had other unfavorable abnormalities (−7, +8, 11q23/MLL gene rearrangement); and 15 patients had other karyotypic abnormalities. Ninety patients were in first complete remission (CR-1) at time of transplant, 58 patients were in CR-2, and 12 patients were in CR-3 or beyond. The characteristics of the study population are summarized in Table 1.

One hundred and fifty-three of the 160 (96%) patients achieved an absolute neutrophil count of 500/mm^3^ on 3 consecutive days at a median of 20 days posttransplant (range: 12–48 days). The seven patients who failed to achieve this level died between days 18 and 86 due to infection complications. One of those seven patients had evidence of leukemia at time of death. One hundred and thirty-three (83%) patients reached a platelet count of 20,000/mm^3^ without transfusions for 7 consecutive days at a median of 20 days (range: 11−99 days). There were 34 relapses by last contact. Day of relapse ranged from 30 to 1485. The median day of relapse was 159 days. Twenty-six of these 34 relapsed patients have died by last contact. There were 66 deaths by last contact; these include relapse related deaths (*n* = 28) and nonrelapse mortality (*n* = 38). Days of death ranged from 13 to 1908 days with a median of 156 days. Median follow-up among the patients who have not died by last contact was 1219 days (range: 182–2487 days). One hundred and twenty-six (79%) patients developed grades 2–4 acute GvHD; 34 (21%) developed grades 3-4 GvHD.

MRD evaluation posttransplant was performed based on our institution standard practice (usually on day +28, day +80–100, and one year after transplant) and patient clinical status and per the discretion of the treating physician.

#### 3.1.1. Effect of Flow Cytometry MRD Results before Transplant on Transplant Outcome

**Figure 1:**
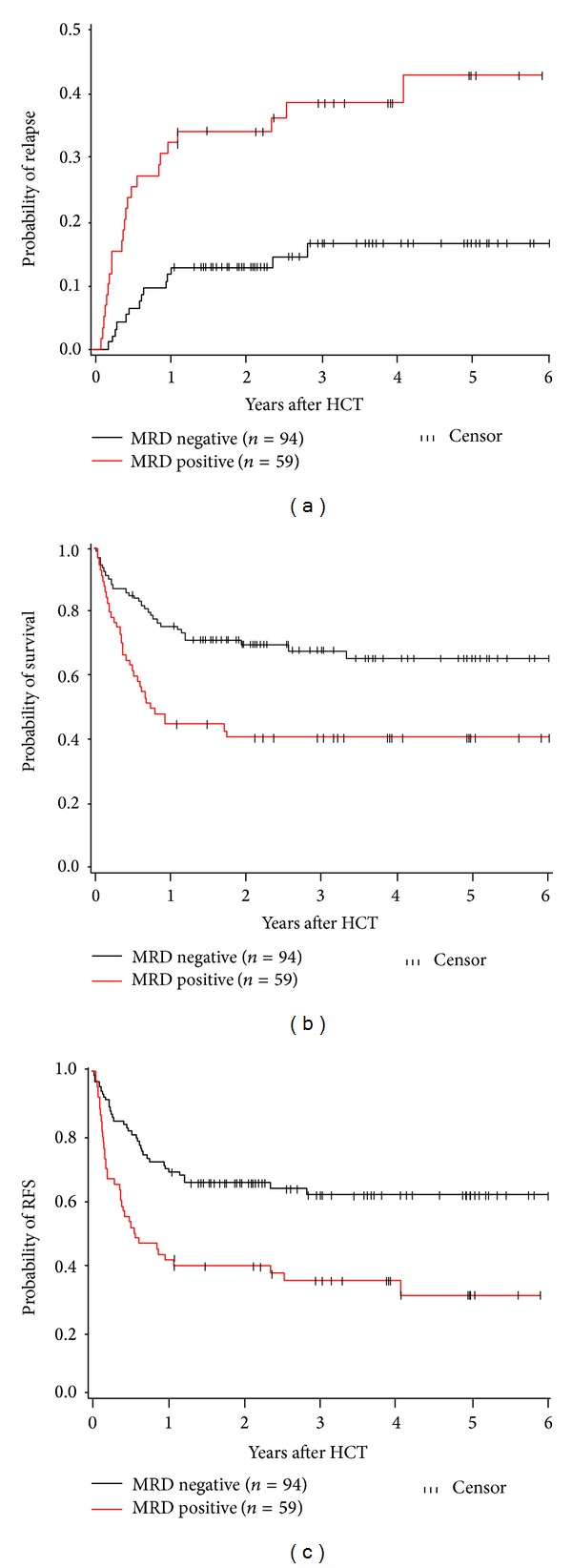
Effect of MRD pre-HCT on transplant outcome. (a) Cumulative incidence of relapse after HCT. One- and 3-year estimates of relapse for patients without MRD pre-HCT were 12% and 17%, respectively, compared to 32% and 38% for patients with MRD pre-HCT. (b) Overall survival after HCT. One- and 3-year estimates of overall survival for patients without MRD pre-HCT were 75% and 68%, respectively, compared to 44% and 40% for patients with MRD pre-HCT. (c) Relapse-free survival. One- and 3-year estimates of relapse-free survival for patients without MRD pre-HCT were 69% and 61%, respectively, compared to 41% and 34% for patients with MRD pre-HCT.

The relationship between the status of MRD before transplant and clinical outcomes was evaluated for 153 patients with an MFC result within 50 days of HCT. Patients with positive MFC results at time of transplant were assigned to the MRD-positive group, while patients with negative MFC results were assigned to the MRD-negative group. All patients had less than 5% bone marrow blasts by morphology and thus met the morphologic criterion for CR. Among 59 patients who were MRD-positive pre-HCT, there were 23 relapses compared to 14 of 94 patients who were MRD-negative pre-HCT (HR = 3.64; 95% CI, 1.87–7.09; *P* = .0001). Sixty-six patients had pre-HCT flow cytometry data and BCR-ABL molecular data. Among patients with PCR data, the risk of relapse for pre-HCT MRD-positive is 3.18 times that of patients who are pre-HCT MRD-negative. After adjusting for BCR-ABL, the HR is essentially unchanged at 2.98. Thirty-five of the 59 MRD-positive patients have died compared to 30 of the 94 MRD-negative patients (HR = 2.39; 95% CI, 1.46–3.90, *P* = .0005). The hazard ratios were qualitatively unchanged after adjusting for the presence of abnormal cytogenetics or positive BCR-ABL by molecular testing. One- and 3-year estimates of relapse for patients without MRD pre-HCT were 12% and 17%, respectively, compared to 32% and 38% among those with MRD pre-HCT, respectively (Figure 1(a)). One- and 3-year estimates of survival for the pre-HCT MRD-negative group were 75% and 68%, respectively, compared to 44% and 40% in the MRD-positive group, respectively (Figure 1(b)). Relapse-free survival estimates at one and three years were 69% and 61%, respectively, in the MRD-negative group and 41% and 34%, respectively, in the MRD-positive group (Figure 1(c)).

In order to see if the degree of MRD among patients correlated with outcome among those who were MRD-positive pre-HCT, we arbitrarily categorized the flow values as less than or equal to 0.01% (*n* = 12), 0.01–0.1% (*n* = 14), 0.1–1.0% (*n* = 16), and 1.0–9.0% (*n* = 11) and greater than 9.0% (*n* = 6). The proportion of posttransplant relapses in these groups were 25%, 50%, 19%, 58%, and 50%, respectively, and the proportion of deaths were 50%, 64%, 50%, 67%, and 67%, respectively. These data provide no convincing evidence to suggest that patients with “more” MRD pretransplant have worse outcome than patients with “less” MRD among those who are MRD-positive, although the numbers are too small to definitively draw a conclusion.

A statistical test for interaction between pre-HCT MRD and T-ALL versus B-ALL yields *P* = .30, although the number of T-ALL is small (*n* = 24), severely limiting the power to detect a meaningful interaction. The risk of relapse was higher among patients with MRD pre-HCT compared to those without MRD among B-ALL patients (HR = 2.87; 95% CI, 1.37–6.02; *P* = .005) and among T-ALL patients (HR = 7.07; 95% CI, 1.31–38.09; *P* = .02). Thus, MRD appears to be correlated with an increased risk of relapse in both B-ALL and T-ALL, but the small sample sizes limit our ability to determine if the effect is larger in the T-ALL patients as compared to the B-ALL patients.

Adjusting for age or CR status at time of transplant has no significant effect on the association between MRD pre-HCT and post-HCT relapse (age *P* = .59; CR *P* = .72).

#### 3.1.2. Effect of Flow Cytometry MRD Results after Transplant on Transplant Outcome

One hundred and forty-four patients had at least one MFC flow sample post-HCT, all of these with at least one sample within 78 days of transplant. One hundred and thirty patients had at least one value within 35 days of transplant, and one hundred and forty patients had at least one value within 50 days of transplant. All of those patients were in remission by marrow morphology (<5% blasts) and with no evidence of extramedullary disease at that time. Modeling post-HCT flow values as a time-dependent covariate showed an increased risk of relapse (HR = 7.47; 95% CI, 3.30–16.92, *P* < .0001) and death (HR = 3.00; 95% CI, 1.44–6.28, *P* = .004) among patients with MRD-positive compared to patients with MRD-negative. If the flow values are restricted to those that occurred within 100 days (to loosely assess the impact of “early” MRD on outcome), the results are qualitatively the same; that is, the relapse rate is higher in MRD-positive patients compared to MRD-negative patients (HR = 4.64; 95% CI, 2.04–10.55, *P* = .0003) as is the rate of mortality (HR = 2.36; 95% CI, 1.14–4.91, *P* = .02). Among the 17 patients with post-HCT MRD-positive, 15 had cytogenetic data. All those 15 patients had normal conventional cytogenetics, but one patient had abnormal FISH. Four patients had BCR-ABL molecular data, of which one was abnormal.

#### 3.1.3. Description of Disease Course after MRD Detection Post-HCT

**Figure 2:**
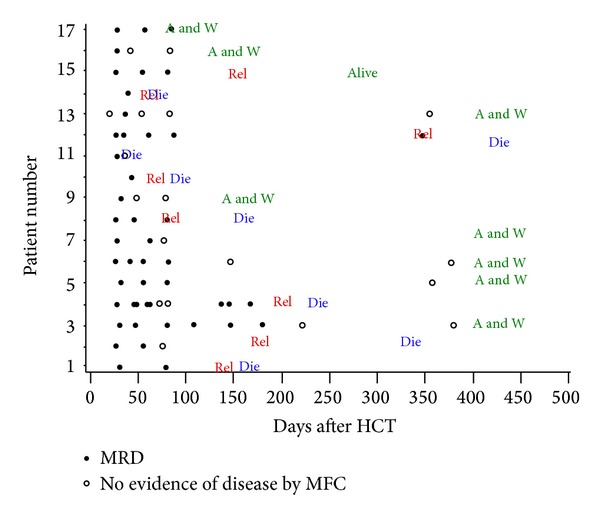
Disease progression after HCT for 17 patients with MRD early post-HCT. Schematic presentation of disease progression of all seventeen patients who were in morphological remission, but with flow cytometry MRD-positive within 50 days posttransplant. Empty circles represent MRD and full circles represent morphological relapse. Rel: relapse; Die: death; A and W: alive and well.

Seventeen patients were in morphological remission, but with evidence of MRD by MFC within 50 days posttransplant. Four of those 17 patients had evidence of MRD in their last marrow evaluation pretransplant. Six patients had MRD level of 0.01% or less, seven patients had MRD level between 0.01% and 0.1%, and four patients had MRD level between 0.1% and 1.0%. Preemptive therapy for patients with MRD-positive after transplant included rapid immunosuppression taper in 3 patients, tyrosine kinase inhibitor (TKIs) in two patients, and rapid immunosuppression taper plus DLI in one patient; the other 11 patients received no intervention. Five of the 11 patients who received no intervention have relapsed, and none of the three patients who had rapid immunosuppression taper had relapsed. The one patient who received DLI had relapsed; a follow-up marrow evaluation 12 days after DLI demonstrated no evidence of disease by morphology, flow cytometry, and cytogenetics; however, the patient developed MRD again two months after DLI, which progressed to overt relapse two months later. Two patients with Ph+ ALL received preemptive therapy with imatinib and dasatinib, respectively. Nonetheless, both patients subsequently relapsed, on day 81 post-HCT and on day 152 post-HCT, respectively. Among the 17 patients who were MRD-positive early (i.e., within 50 days) post-HCT, 8 patients relapsed during the follow-up period and 7 of them died. Seven patients who were MRD-positive early after transplant converted to MRD-negative and were alive with no evidence of relapse at last contact at median of 726 days after transplant. MFC levels for these patients were 0.0014% to 0.8%, and MRD cleared between day 35 and day 357 post-HCT. Three of the patients who converted to MRD-negative had rapid taper of immunosuppression and four had no intervention. All seven patients who cleared their MRD and have not relapsed developed grade II-III acute GVHD, and three developed chronic GVHD. Among patients with early posttransplant MRD who eventually relapsed, two patients cleared their MRD prior to progressing to overt relapse. Disease progression and outcome after HCT for all 17 patients with MRD-positive early post-HCT is shown in Table 2 and Figure 2.

#### 3.1.4. Combined Effects of Pre- and Post-MRD Detection

Combining the early post-HCT data with the pre-HCT flow status revealed that MRD positivity at any time is associated with worse outcome relative to patients who were MRD-negative before and after HCT. With post-HCT MRD status modeled as a time-dependent covariate for the post-HCT closest to day 28, patients who were MRD-negative pre-HCT and became MRD-positive post-HCT had an increased risk of relapse compared to negative/negative patients (HR = 8.08; 95% CI, 1.79–36.36, *P* = .007). Patients who were MRD-positive pre-HCT but MRD-negative post-HCT were also more likely to relapse relative to negative/negative (HR = 3.61; 95% CI, 1.69–7.73, *P* = .0009) as were patients who were MRD-positive pre-HCT and MRD-positive post-HCT (HR = 5.86; 95% CI, 2.39–14.39, *P* = .0001). There were only 3 patients who were MRD-negative pre-HCT but became MRD-positive at some point post-HCT. Qualitatively similar results were seen for overall mortality. In particular, negative/positive versus negative/negative yielded HR = 3.75 (95% CI, 0.89–15.81, *P* = .07); positive/negative yielded HR = 2.41 (95% CI, 1.41–4.12, *P* = .001); and positive/positive yielded HR = 2.87 (95% CI, 1.35–6.12, *P* = .006).

## 4. Discussion

In this study, we investigated the prognostic value of MRD, as detected by MFC before and after myeloablative allo-HCT in a large cohort of patients with ALL. A number of reports have shown that MRD prior to HCT is a predictive factor for relapse after HCT [8–16, 29, 30, 32–34]. In these studies, the EFS for the MRD-negative groups prior to HCT was often more than 70%, while for the MRD-positive group the EFS fell to approximately 17–50%. The majority of those studies evaluated MRD by Ig/TCR gene rearrangement, included relatively small numbers of ALL patients, and evaluated the effect of MRD prior to transplant. Only a few studies have demonstrated the impact of MFC-based MRD screening in the allogeneic transplant setting. In one study, bone marrow samples were prospectively taken from 24 ALL patients at certain time points before and after transplantation. The data showed that pretransplant MRD detected by MFC was a significant predictor of outcome, and indicated that MRD monitoring after HCT could be used to identify patients with a high risk of relapse [29]. Zhao et al. also demonstrated that a MRD-positive after HCT is correlated with poor EFS and high cumulative incidence of relapse (CIR) in both high-risk and standard-risk groups of ALL patients [30]. The results of our study support those prior findings in a large cohort and demonstrate the importance of MFC as a tool to evaluate ALL MRD before and after transplant. MFC is less labor intensive and more widely available than PCR, and the results of our study demonstrate that MFC may be used to risk stratify ALL patients before and after HCT.

The data presented in this retrospective analysis support several conclusions.* First*, patients with ALL who were in CR without flow cytometric evidence of MRD before myeloablative allogeneic HCT had a better outcome than patients with evidence of MRD.* Second*, patients with evidence of MRD after transplant (and in particular, early after transplant) had significantly worse outcomes than patients without evidence of MRD after transplant.* Third*, not all patients who became MRD-positive after transplant relapsed, with approximately one-third reverting to MRD-negative status. As shown in Table 2, patient number 3 had no evidence of relapse more than 4 years after transplant, patient number 5 had no evidence of relapse more than 3 years after transplant, patient number 6 had no evidence of relapse 2 years and 8 months after transplant, patient number 7 had no evidence of relapse 2 years after transplant, and patient number 13 had no evidence of relapse 1 years and 7 months after transplant. Thus, four out of the seven patients who converted from MRD-positive to MRD-negative after transplant had been in remission for at least two years posttransplant, and one patient had been in remission for more than 18 months posttransplant. Patients number 9, number 16, and number 17 had short follow-up of less than a year, and therefore we cannot comment on their long term disease status after transplant. Based on this data, at least four of seventeen patients (24%) with MRD-positive before day 50 post-HCT have converted to MRD-negative and had been in remission for 2 years or more after transplant. Among the seven patients in our cohort who converted their MRD status, three had rapid immunosuppression taper and four had no intervention. Clearing of MRD after allo-HCT may be a result of graft-versus-leukemia effect. This potential mechanism is supported by the observation that all seven patients in our cohort who did not relapse and had no evidence of MRD developed graft-versus-host disease.

It is difficult to draw firm conclusions regarding the association between level of MRD and outcome (i.e., whether patients with higher MRD levels have a worse outcome than those with lower levels). For pretransplant MRD, our data support the notion that not the MRD level but the presence of MRD increases the probability of relapse with equal proportion of posttransplant relapses (50%) for pretransplant MRD of 0.01%–0.1% or MRD of more than 9%. However, the situation may be different for posttransplant MRD. Among 17 patients with posttransplant MRD-positive within 50 days after transplant, six patients had MRD level of 0.01 or less, four of whom (all with MRD level below 0.01%) did not develop relapse during the follow-up period (2 years or more for two patient, and less than one year for two patients). On the other hand, three of four patients with MRD higher than 0.1% have relapsed (the fourth patient with MRD level of >0.1% had early death due to infection and treatment related complications). Despite the small numbers, these results may support correlation between posttransplant MRD level and outcome. One may speculate that lower risk of relapse with lower level of MRD after transplant may be due to graft versus leukemia effect, which is more effective when the disease burden is low. A more definitive examination of the correlation between pre- and posttransplant MRD level and outcome will require a larger number of MRD-positive patients.

This study has several limitations. The data were collected retrospectively, the patient population is heterogeneous, patients were treated according to a variety of protocols with different treatment strategies, and methods and timing of follow-up were not standardized. Despite these limitations, we believe that this study provides strong evidence that both pre- and post-HCT MRD, detected by MFC, are associated with increased relapse and decreased survival after transplant.

Determination of MRD, early after induction and/or after consolidation chemotherapy, has proven useful to predict relapse and poor outcome in ALL patients and may help in identifying patients who require allogeneic HCT for treatment intensification [5]. Our findings suggest that detection of MRD by MFC at the time of HCT or early after HCT defines a population of ALL patients who are at higher risk for adverse outcome, even after adjusting for other factors that influence post-HCT outcome. These findings support the use of pre- and post-HCT MRD assessment by MFC for risk stratification of post-HCT outcome. Furthermore, these findings provide the rationale for future studies to test whether HCT outcome of MRD-positive ALL patients can be improved through MRD-stratified interventions, for example, preemptive treatment after HCT.

## Figures and Tables

**Table 1:** Patient characteristics.

Characteristics	Number (%)
Number of patients	160
Median age (range), years	24.6 (0.6–61.8)
Age < 18 years	62 (39)
Gender (male/female)	99/61
Lineage type	
B-ALL	134 (84)
T-ALL	24 (15)
Biphenotypic leukemia	2 (1)
Cytogenetic subgroup	
Normal	99 (62)
t(9;22)	31 (19)
Other unfavorable abnormalities (−7, +8, 11q23)	15 (9.5)
Other karyotypic abnormalities	15 (9.5)
Disease status at time of HCT	
CR1	90 (56)
CR2	58 (36)
≥CR3	12 (8)
Donor Origin	
Related	51 (32)
Unrelated	109 (68)
Donor type (*n* = 125, excluding cord blood)	
Match	102 (64)
Mismatch	23 (14)
Graft source	
Bone marrow	59 (37)
Mobilized blood	66 (41)
Cord blood	35 (22)
TBI based conditioning	142 (89)
GVHD prophylaxis	
Tacrolimus based regimen	100 (62%)
CSP based regimen	30 (19%)
Other	30 (19%)

**Table 2:** MRD after HCT in 17 patients who were MRD-positive before day 50 post-HCT.

Patient	Days and values of MRD (%)											Day of relapse	Day of death	Day of last contact	Treatment for MRD
1	32	80										144	164		None
	0.01	0.03													
2	27	55	76									173	337		None
	0.004	0.01	0.0												
3	32	48	81	109	147	181	222	732	930	1294	1428	N/A	N/A	1707	Rapid IS taper
	0.03	0.1	0.18	0.07	0.10	0.02	0.00	0.00	0.00	0.00	0.00				
4	28	46	49	60	62	74	82	139	145	168		199	237		Rapid IS taper/DLI
	0.3	0.5	0.019	0.5	0.13	0.00	0.00	0.15	0.1	1.8					
5	33	56	80	357								N/A	N/A	1172	Rapid IS taper
	0.009	0.008	0.001	0.00											
6	28	42	56	82	147	377						N/A	N/A	1001	Rapid IS taper
	0.02	0.02	0.04	0.04	0.00	0.00									
7	28	63	77									N/A	N/A	726	None
	0.0014	0.005	0.00												
8	27	46	81									81	156		Imatinib
	0.15	0.02	6.5												
9	33	48	82									N/A	N/A	149	None
	0.04	0.00	0.00												
10	44											65	90		None
	0.04														
11	28	35										N/A	39		None
	0.8	0.0													
12	28	35	61	88	347							347	629		None
	0.1	0.06	0.04	0.07	7.3										
13	21	37	55	83	355							N/A	N/A	579	None
	0.00	0.03	0.00	0.00	0.00										
14	41											61	65		None
	0.04														
15	28	56	81									152	N/A	265	Rapid IS taper/Dasatinib
	0.3	0.02	0.02												
16	28	43	85									N/A	N/A	134	None
	0.002	0.00	0.00												
17	29	57	84									N/A	N/A	87	None
	0.003	0.001	0.002												

IS: immunosuppression.
